# The Role of Radiotherapy Among the Therapeutic Options for Castleman’s Disease

**DOI:** 10.4274/tjh.2013.0164

**Published:** 2014-06-10

**Authors:** Feryal Karaca, Çiğdem Usul Afşar, Erkut Erkurt, Hasan Suat Arslantaş, Elif Çalış, Berna Totan Ateş, Emine Bağır, Melek Ergin, Semra Paydaş

**Affiliations:** 1 Çukurova University Faculty of Medicine, Department of Radiation Oncology, Adana, Turkey; 2 Çukurova University Faculty of Medicine, Department of Medical Oncology, Adana, Turkey; 3 Çukurova University Faculty of Medicine, Department of Pathology, Adana, Turkey

**Keywords:** Castleman’s disease, Radiotherapy, Unicentric

## TO THE EDITOR

Castleman’s disease, also known as giant lymph node hyperplasia or angiofollicular lymph node hyperplasia, was first defined in 1956 by Castleman and his colleagues. It usually appears as a mediastinal, cervical, mesenteric, or retroperitoneal mass [1]. Castleman’s disease is divided into 2 different types, hyaline vascular or plasma cell, based on histopathological features [2]. Cases with mixed histological features have also been reported [3,4]. There are 2 clinical types: localized or multicentric [3]. There is also another form associated with HIV positivity.

**Figure 1:**
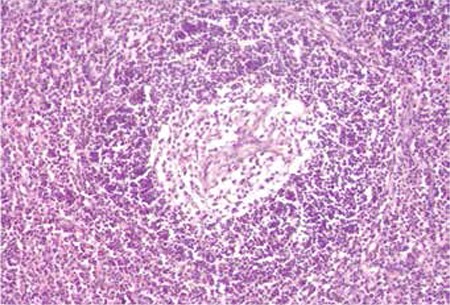
Appearance under light microscope with H&E staining and 20x magnification.

Castleman’s disease may be accompanied by malignant lymphoma, vascular neoplasm, follicular dendritic cell tumor, or Kaposi’s sarcoma [5,6,7]. The localized form is usually asymptomatic; surgical removal of the lesion is curative and it does not progress to lymphoma or other diseases. However, the multicentric form usually appears as the plasma cell type, leads to systemic complaints, and is treated medically [8,9]. Multicentric Castleman’s disease is quite aggressive and may progress to non-Hodgkin’s lymphoma. The localized hyaline vascular type is the most common and it clinically presents with abdominal, mediastinal, and cervical masses. We present here a 15-year-old female patient with a neck mass for 6 months who presented with enlargement of the mass for the last 2 months and shortness of breath at night. On physical examination, palpation revealed a hard, fixed, and indeterminably circumscribed mass extending to the mediastinum. Laboratory tests showed no cytopenia. Upon magnetic resonance imaging of the soft tissue of the neck and thorax, a soft mass of 9x5x6 cm was detected. The patient underwent lymph node excision for diagnostic purposes. The pathological report revealed hyaline vascular-type Castleman’s disease (Figure 1). The patient was considered to be inoperable since the mass was large and fixed to the surrounding tissues. After giving a total dose of 3060 cGy external radiotherapy (RT) to the left neck and supraclavicular lymph nodes in 17 fractions of 180 cGy, a total dose of 4500 cGy curative RT was given by boost to the involved lymph nodes. On the control computed tomography examination, in comparison to the previous imaging, partial regression was observed and the mass was 5 cm in the axial plane. The patient has been followed for 18 months and the disease is still stable. Systemic inquiry of the patient revealed no symptoms. In Castleman’s disease of the localized form, prognosis is excellent with surgical resection of well-circumscribed neck masses [3]. However, the present patient had no chance of surgery.

Adjuvant therapy is needed for the cases in which resection is not available or incomplete [10]. A study performed at the MD Anderson Cancer Center, including 22 patients, investigated the role of RT in unicentric and multicentric disease. The paper reported that RT provided clinical response and cure in the selected patients [11]. Cure has been achieved after resection in all cases in the literature [12]. Unlike the localized form, there is no consensus on the treatment of the multicentric form. Chemotherapy and corticosteroid therapy are given in addition to surgery; however, response is variable and prognosis is poor. 

In conclusion, Castleman’s disease should be considered in the front line of possibilities in the differential diagnosis of mediastinal masses. Complete resection by surgery results in excellent early and late outcomes. As in the present case, RT is an appropriate option for masses that cannot be resected by surgery. RT can be a definitive treatment modality of unicentric Castleman’s disease with a good control rate and few complications, as seen in a review of the literature [13]. Three-dimensional conformal RT and intensity modulated radiation therapy can also be used in unresectable unicentric cases [14]. The overall response rate in localized Castleman’s disease is about 70% (complete response rate is about 44% and partial response rate is 29%), and almost all responding patients sustain stable or remission status. Only a few cases show no response to RT. As the techniques of RT have developed, it is possible to deliver high-dose radiation to target tumors with minimal complications [13].

## CONFLICT OF INTEREST STATEMENT

The authors of this paper have no conflicts of interest, including specific financial interests, relationships, and/ or affiliations relevant to the subject matter or materials included.
